# Fetal Hydantoin Syndrome: A Case Report

**DOI:** 10.7759/cureus.49663

**Published:** 2023-11-29

**Authors:** Sanchit Aggarwal, Manidipa Barman, Binita Poudel, Kamal Joshi, Risha Devi, Poonam Singh, Mayank Priyadarshi, Suman Chaurasia, Sriparna Basu

**Affiliations:** 1 Paediatrics, All India Institute of Medical Sciences, Rishikesh, IND; 2 Neonatology, All India Institute of Medical Sciences, Rishikesh, IND

**Keywords:** women with epilepsy, antiepileptic drugs, phenytoin embryopathy, fetal embryopathy, fetal hydantoin syndrome

## Abstract

Epilepsy is not a common cause of morbidity in pregnancy. It has widespread effects on maternal and fetal health necessitating adequate control of seizures. Many anti-seizure medications (ASM) have teratogenic effects on the fetus. We report a case of severe fetal hydantoin syndrome resulting in life-threatening major congenital anomalies. The mother was on phenytoin for the last three years and the pregnancy was not registered. We discuss various features of fetal hydantoin syndrome and the ideal management of epilepsy in pregnancy in brief.

## Introduction

Approximately three to five in 1,000 pregnant females have epilepsy [1]. These prospective mothers need to continue anti-seizure medications (ASM) in order to avoid the harmful effects of seizures on themselves and the fetus. These include the risk of abortion, gestational hypertension, preeclampsia, preterm delivery and maternal death [2-4]. Moreover, anti-epileptic drugs (AEDs) themselves might prove harmful to the fetus by causing congenital malformations and affecting childhood cognition [5,6]. Such an association between AED usage in pregnancy and teratogenicity was first reported with the use of mephenytoin, and subsequently, carbamazepine, valproate, barbiturates and phenytoin have been implicated in causing major congenital malformations [7]. We describe a case of severe fetal hydantoin syndrome, which is a characteristic pattern of congenital malformations resulting from antenatal maternal intake of phenytoin [8].

## Case presentation

A 26-year-old gravida 2 (G2P1L1) unbooked mother presented to our institute at 29 weeks + 2 days of gestation with severe and persistent antepartum hemorrhage. The pregnancy resulted from a spontaneous conception. On history and assessment, it was found that the lady was on a daily phenytoin dose of 300 mg, in three divided doses, for the last three years. The medication was started in view of persistent seizures during the post-partum period of her previous pregnancy three years ago. The cause of antenatal hemorrhage was ascertained to be grade 4 placenta praevia. The patient did not have any antenatal visits in the first trimester and had no fever, rash, or teratogen exposure besides daily phenytoin. There was no antenatal workup to rule out any high-risk factors or ultrasonography. She had only one antenatal visit in the second trimester and did not receive any supplements or recommended vaccines. There was no other significant medical history. The patient could not receive antenatal corticosteroids or magnesium sulphate prior to the delivery.

**Figure 1:**
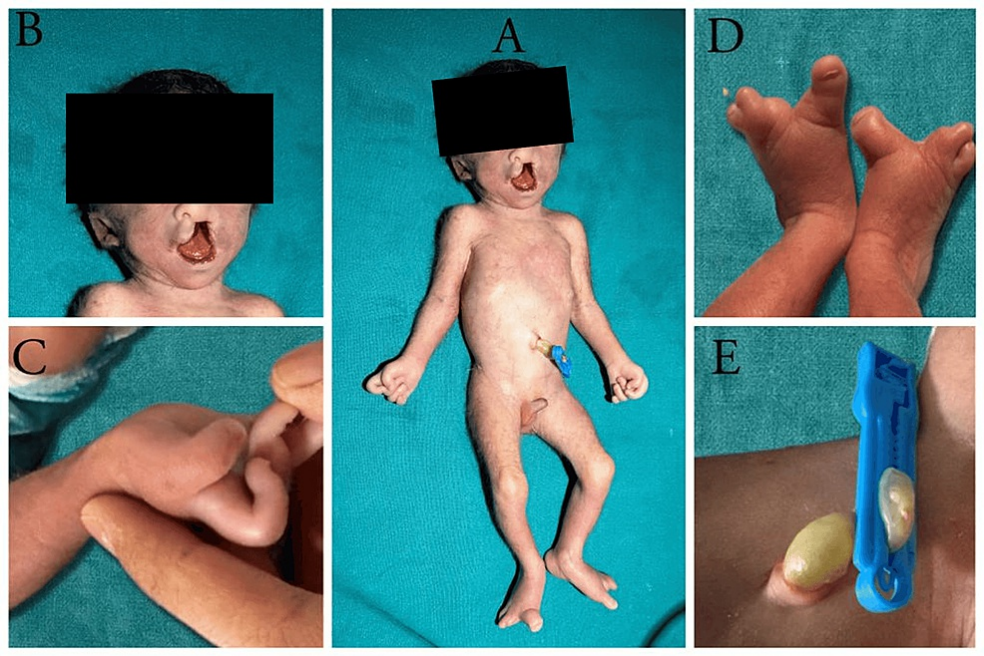
Clinical photographs of the patient showing characteristic features of fetal hydantoin syndrome: (A) hypertelorism, broad nasal bridge, cleft lip and low-set ears. (B) Hypoplastic distal phalanges in the right hand with contractures. (C) Oligosyndactyly. (D) Absent distal phalanges in bilateral feet. (E) Single umbilical artery.

The neonate was delivered by an emergency lower-segment caesarean section and did not cry at birth. Adequate resuscitative measures were taken, and the baby was intubated in view of absent respiratory efforts. Apgar scores were 3 and 4 at one and five minutes of life, respectively. The baby had no spontaneous efforts and required 100% FiO2 on mechanical ventilation and a peak inspiratory pressure of 20 cm H2O with a positive end-expiratory pressure of 5 cm of H2O on the assist control mode of mechanical ventilation. The birth weight was documented to be 1,091 grams. On clinical examination, the baby had multiple dysmorphic features (Figure 1). There was microcephaly, hypertelorism, complete cleft lip and palate, low-set ears, finger contractures bilaterally, oligosyndactyly in bilateral lower limbs and a two-vessel umbilical cord. Echocardiography was performed in view of a pansystolic murmur auscultated at the apex and was suggestive of a large ventricular septal defect with a double outlet right ventricle. The parents were explained about the neonate’s condition, and they made an informed decision to not escalate treatment and not resuscitate the baby if needed again. The neonate went into bradycardia and later expired after three hours of life.

## Discussion

The term 'fetal hydantoin syndrome' was first proposed by Hanson and Smith in 1975 for a constellation of birth defects seen in neonates born to mothers with a history of antenatal phenytoin intake [9]. They described craniofacial anomalies, microcephaly and hypoplasia of the distal phalanges in these neonates. Subsequently, it was reported that antenatal intake of phenytoin leads to characteristic facies with a short neck, low-set ears, cleft lip and palate, deep nasal bridge and hypoplastic distal phalanges of both upper and lower limbs [10]. Microcephaly, growth restriction, impaired cognition, hernias and hypospadias have also been reported in a few patients [9]. Congenital heart diseases have also been reported in such neonates and include valvular stenosis, coarctation of the aorta and ventricular septal defects [11].

The teratogenicity of phenytoin has been speculated to be due to multiple factors, but no conclusive pathophysiology of these malformations has been outlined yet [8]. A few authors have suggested that decreased levels of folate lead to the syndrome [12,13]. Genetic factors like defects in the detoxification of phenytoin arene oxide, complex interactions between multiple AEDs, maternal age and period of gestation have also been implicated in contributing to phenytoin teratogenicity [8].

The International League Against Epilepsy recommends that all women with epilepsy (WWE) be managed in close association with a neurologist and obstetrician [14]. Adequate counselling regarding the need to control seizures, their adverse effects and the teratogenicity of AEDs should be done. Therapeutic drug monitoring in the antenatal period is a must when phenytoin and phenobarbitone are being used and should be done monthly. Monotherapy should be followed as closely as possible. According to the CDC, the use of pre-conception folate of 4 mg per day should be favoured, beginning one month prior to conception in planned pregnancies and all WWE in childbearing age and continuing for three months into pregnancy. The league also performed a questionnaire-based global survey of guidelines on the management of epilepsy in pregnant females and found that almost two-thirds of their national chapters had guidelines for such patients, although a significant proportion of these were last reviewed prior to 2014 [15]. Also, there was a wide variation in the folate dose recommended for WWE.

The current case becomes pertinent in a hilly terrain region of a low-middle-income country as the WWE described in our case continued her AED without supervision or therapeutic level monitoring, and folate supplements were not received due to a lack of antenatal follow-up. These factors could have resulted in the severe form of fetal hydantoin syndrome seen in the neonate.

## Conclusions

The case report elucidates clinical findings of a severe case of fetal hydantoin syndrome in a neonate born to a mother with poor antenatal follow-up and highlights the importance of close follow-up during pregnancy, multiple contacts with the antenatal clinic, appropriate use of supplements, adherence to specialist advice in cases of comorbidities like epilepsy and avoidance of potentially teratogenic medications. Clinicians should also be aware of such risks, ensure antenatal measures to prevent such malformations and identify these signs in neonates when such cases present.

## References

[1] Viinikainen K, Heinonen S, Eriksson K, Kälviäinen R (2006). Community-based, prospective, controlled study of obstetric and neonatal outcome of 179 pregnancies in women with epilepsy. Epilepsia.

[2] MacDonald SC, Bateman BT, McElrath TF, Hernández-Díaz S (2015). Mortality and morbidity during delivery hospitalization among pregnant women with epilepsy in the United States. JAMA Neurol.

[3] Laganà AS, Triolo O, D'Amico V (2016). Management of women with epilepsy: from preconception to post-partum. Arch Gynecol Obstet.

[4] Viale L, Allotey J, Cheong-See F (2015). Epilepsy in pregnancy and reproductive outcomes: a systematic review and meta-analysis. Lancet Lond Engl.

[5] Koo J, Zavras A (2013). Antiepileptic drugs (AEDs) during pregnancy and risk of congenital jaw and oral malformation. Oral Dis.

[6] Christensen J, Grønborg TK, Sørensen MJ, Schendel D, Parner ET, Pedersen LH, Vestergaard M (2013). Prenatal valproate exposure and risk of autism spectrum disorders and childhood autism. JAMA.

[7] Morrow J, Russell A, Guthrie E (2006). Malformation risks of antiepileptic drugs in pregnancy: a prospective study from the UK Epilepsy and Pregnancy Register. J Neurol Neurosurg Psychiatry.

[8] Oguni M, Osawa M (2004). Epilepsy and pregnancy. Epilepsia.

[9] Hanson JW, Smith DW (1975). The fetal hydantoin syndrome. J Pediatr.

[10] Singh R, Kumar N, Arora S, Bhandari R, Jain A (2012). Fetal hydantoin syndrome and its anaesthetic implications: a case report. Case Rep Anesthesiol.

[11] Godbole KG, Gambhir PS, Deshpande AS, Kurlekar SU, Phadke MA (1999). Fetal hydantoin syndrome with rheumatic valvular heart disease. Indian J Pediatr.

[12] Speidel BD, Meadow SR (1972). Maternal epilepsy and abnormalities of the fetus and newborn. Lancet Lond Engl.

[13] Hernández-Díaz S, Werler MM, Walker AM, Mitchell AA (2000). Folic acid antagonists during pregnancy and the risk of birth defects. N Engl J Med.

[14] Tomson T, Battino D, Bromley R, Kochen S, Meador K, Pennell P, Thomas SV (2019). Management of epilepsy in pregnancy: a report from the International League Against Epilepsy task force on women and [regnancy. Epileptic Disord.

[15] Tomson T, Battino D, Bromley R, Kochen S, Meador KJ, Pennell PB, Thomas SV (2020). Global survey of guidelines for the management of epilepsy in pregnancy: a report from the International League Against Epilepsy task force on women and pregnancy. Epilepsia Open.

